# Linear growth in young people with restrictive eating disorders: “Inching” toward consensus

**DOI:** 10.3389/fpsyt.2023.1094222

**Published:** 2023-03-03

**Authors:** Amanda E. Downey, Alexis Richards, Anna B. Tanner

**Affiliations:** ^1^Department of Pediatrics, University of California, San Francisco, San Francisco, CA, United States; ^2^Department of Psychiatry and Behavioral Sciences, University of California, San Francisco, San Francisco, CA, United States; ^3^Department of Pediatrics, Emory University, Atlanta, GA, United States; ^4^Accanto Health Perimeter Center East, Dunwoody, GA, United States

**Keywords:** eating disorders, anorexia nervosa, ARFID, growth, malnutrition

## Abstract

**Background:**

While the assessment of acute medical stability in patients with eating disorders should never be minimized, careful attention toward other specific age-related consequences of malnutrition can improve psychological outcomes and reduce long-term, potentially irreversible medical complications, like linear growth impairment.

**Review:**

While the impact of malnutrition on linear growth is widely recognized, emerging data highlight consensus in several key areas: the time from onset to time of diagnosis, age at illness onset, pubertal stage at illness onset, and adequacy of weight restoration to achieve catch-up growth. This review provides concrete and actionable steps to help providers identify and explore deviations in expected growth and development while prioritizing early and aggressive weight restoration to provide the best opportunity for catch-up linear growth in patients with eating disorders.

**Conclusion:**

The impact of restrictive eating disorders on growth and development cannot be overstated, particularly in pre- and peripubertal patients. While many consequences of malnutrition are reversible, the loss of genetic height potential may prove irreversible without early and aggressive weight restoration.

## Introduction

Eating disorders are serious biopsychosocial disorders with medical complications that can affect every organ system and psychological impairment that can derail every facet of a person’s life (1). Given high mortality rates, much attention has been paid to the medical management of eating disorders, particularly life-threatening medical complications. While the assessment of acute medical stability should never be minimized, careful attention toward other specific age-related consequences can improve psychological outcomes and reduce long-term, potentially irreversible medical complications.

Children and adolescents should be growing (2). Compared to adult patients with eating disorders, younger patients experiencing energy deficits may be slower to exhibit traditional signs of malnutrition, like vital sign instability, and may instead display slowed growth (3). Linear growth is one such important metric of growth and may also be referred to as height or crude height (4). Indeed, growth stunting has been called “covert tissue injury” to align with the other complications seen in all organ systems (5). The loss of potential linear growth and the inability to appropriately mineralize and strengthen bone during critical periods of development are potentially irreversible consequences of malnutrition (6). By diligently monitoring for growth disruptions, pediatric providers play a critical role in the identification of young people struggling with eating disorders and subsequently in monitoring their growth and development as a key member of a multidisciplinary eating disorders team (7). It is important to note that clinicians should screen for other potential etiologies of growth impairment, including growth hormone deficiency, celiac disease, inflammatory bowel disease, and genetic disorders, among others (8).

Tracking growth and physical development is a primary focus of annual physical exams for children and adolescents in the United States (8). Normal growth follows predictable patterns (2). Children will establish a weight percentile and height percentile in early childhood and track along each of those percentiles until growth is complete. Deviations from expected patterns are cause for concern and further investigation for an underlying disorder is warranted (2). In young people with malnutrition, deviations or percentile drops in weight occur first, followed by deviations in height (2). Because children and adolescents should be growing–cessation or slowing of expected growth patterns are an important cause for concern, even if frank weight loss is absent (9). Acquisition of predicted height potential is a marker of wellbeing (8, 10), and conversely, failure to achieve predicted linear growth potential is a marker of reduced neurodevelopmental and cognitive function and poses elevated risk of chronic disease in adulthood (10). Indeed, linear growth failure has been identified as a major global health priority (11).

Previous studies of stunted linear growth support the possibility of catch-up growth under ideal conditions. Animal models employing growth-inhibiting conditions, such as nutritional deficiencies (12), hypothyroidism (13), and glucocorticoid excess (14), show functional changes in the growth plates which inhibit cellular proliferation and growth. When these conditions resolve, growth plates show accelerated proliferation resulting in catch-up linear growth. Studies replicated in children with Crohn’s disease (15) and celiac disease (16) support the findings from animal models and find adequate catch-up linear growth when growth-promoting conditions are restored.

Previous studies examining linear growth in patients with anorexia nervosa (AN) are limited by small sample sizes, failure to include final adult height, and/or failure to incorporate premorbid height data, yielding inconclusive or even conflicting accounts of this complication of malnutrition (9, 17–20). In a prospective, observational study of 255 female adolescent patients with anorexia nervosa, final adult height was significantly lower than expected, despite achieving anticipated premorbid height (21). Despite even smaller sample sizes, similar height stunting has been found in male patients with anorexia nervosa (22). In a meta-analysis on the effects of restrictive eating disorders on growth and puberty by Neale et al. (20) growth delay was common in patients with restrictive eating disorders, though catch-up growth was observed. In some cases, catch up growth was suboptimal (20). The authors found moderate evidence that weight gain was associated with catch-up growth (20). While the impact of malnutrition on linear growth merits further study, emerging data highlight consensus in several key areas which is of high value to clinicians: the time from onset to time of diagnosis, age at illness onset, pubertal stage at illness onset, and adequacy of weight restoration to achieve catch-up growth.

## Predicting linear growth

The corrected mid–parental height method is anevidence-based way to approximate genetic potential for linear growth and common way to evaluate the efficacy of growth-promoting interventions (23, 24). Like the calculation of weight restoration goals, the thoughtful identification of genetic height potential can inform the pace and intensity of treatment. Unlike weight restoration, there is a finite developmental period where “catch-up” growth is possible. Patients with more significant growth delays and/or less time for catch-up growth may benefit from intensive treatment that supports more aggressive weight restoration and resumption of growth. Because annual visits with pediatric providers are standard, significant delays in the identification of weight or height suppression may result in irreversible consequences. For any deviations in growth, expeditious evaluation, treatment, and close follow up for resolution of growth concerns is crucial. Providers can utilize the following formulas, introduced by Tanner in 1970, for calculating mid-parental height (MPH) (23).

• Mid-parental height for females = (Father’s height (in) + Mother’s height (in)–5)/2.
• Or (Father’s height (cm) + Mother’s height (cm)–13)/2.
• Mid-parental height for males = (Father’s height (in) + Mother’s height (in) + 5)/2.
• Or (Father’s height (cm) + Mother’s height (cm) + 13/2.
• Predicted range ± 3 inches (8.5 cm).

While these formulas have not been specifically validated for use in those with restrictive eating disorders and may be misleading in the case of extreme parental height, they can provide useful context for tracking linear growth.

## Pathophysiology of linear growth and growth impairment in restrictive eating disorders

Alterations in the standard child and adolescent hormonal milieu provides a mechanistic explanation for growth impairment in patients with restrictive eating disorders, though the exact etiology is likely multifactorial and not completely understood. Pubertal development and linear growth is typically accompanied by distinct changes in growth hormone (GH) and insulin-like growth factor (IGF), which ultimately creates an anabolic environment, stimulating longitudinal bone growth (25). In adolescents with AN, alterations in the GH-IGF axis demonstrate GH resistance and concurrent impaired linear growth (25–27). Additionally, IGF-I deficiency correlates with low BMI in AN (25, 26, 28). Malnutrition-induced decreases in T4 (thyroxine) and T3 (triiodothyronine) lowers resting energy expenditure, thus preserving energy for vital functions (29). The hypercortisolemia seen in young people with AN stimulates osteoclasts, causes further dysregulation of the GH-IGF axis, and impairs renal and gastrointestinal absorption of calcium, further yielding alterations of linear height trajectory (29). Combined, these effects yield alterations in linear height trajectory.

## Significance of illness onset and puberty

Hypogonadotropic hypogonadism resulting from malnutrition dramatically alters pubertal development, the growth spurt, and menarche. Estradiol and testosterone levels in adolescents with anorexia nervosa are markedly lower than same-gender nourished controls (30–32). A paucity of sex hormones leads to decreased serum levels of IGF-I, yielding diminished stimulation of longitudinal bone growth (28, 33). While weight gain can accelerate growth, the time to reverse the effects of malnutrition on pubertal development takes time. In one cohort, the time between peak growth velocity and menarche was significantly longer than in healthy controls (34, 35). In another study, weight gain to the prepubertal growth trajectory and completion of the pubertal growth spurt were necessary to reach menarche in those with an eating disorder and primary amenorrhea (36). While the progression through pubertal development was sequentially identical to healthy controls, the pace was slower (35). Indeed, those patients presenting with eating disorders aged <13 years or less than 1 year after menarche showed more severe linear growth impairment compared to those who presented at older age, which may be explained by older patients having already progressed through their linear growth spurt with adequate energy stores prior to onset of the eating disorder (5). While the development of an eating disorder at younger age seemingly implies more time for catch up growth with associated delays in skeletal maturity, studies instead show trends toward irreversible growth stunting (5, 18, 21). Overall, younger age at diagnosis portends higher risk for loss of linear height, although delayed skeletal maturity in this population *may* confer longer duration for catch-up growth. Simply put, as time progresses with illness, growth plates can mature and fuse despite malnutrition, which may result in less growth than would be genetically predicted. These findings underscore the need for early diagnosis and aggressive nutritional rehabilitation to reestablish premorbid growth trajectories, with additional adjustment for the expected weight gain during normal growth and development.

## Significance of illness onset and time to diagnosis

Shorter time to diagnosis and expediency of weight restoration predicts positive short and long-term outcomes, including remission from illness and global improvement in eating disorder pathology (37–41). Multiple studies attempt to quantify the impact of duration of illness prior to diagnosis or intake. One multi-site study of outpatient eating disorder programs showed a mean duration of illness of 5.7–18.6 months prior to intake, and found that a shorter duration of illness prior to intake predicted improved weight outcomes at 1 year (42). Another study comparing a standardized care path in those with nutritional insufficiency presenting to an academic medical center versus a community hospital found illness durations of 1.3 years compared to 1.2 years, respectively (43). A meta-analysis of 459 subjects revealed younger age and longer duration of illness as risks for linear growth stunting (20). Time to diagnosis is not clearly defined in these studies and factors which may have caused diagnostic delay are not fully explored.

One retrospective study aimed to compare the time to diagnosis as characterized by two distinct measurements: time of parental identification of symptoms to diagnosis versus time of noted deviation in weight growth curve to diagnosis (44). This study confirms anecdotal reports that those with rapid weight loss come to medical attention more quickly, resulting in earlier diagnosis and treatment initiation (44). Those with less stark physical changes, including weight stagnation, went undetected longer – suggesting less recognition by caregivers and less clinician urgency in pursuing further evaluation, despite deviation from expected growth trajectory. Importantly, almost half of this sample of patients could have been identified with deviations in the growth curve at a median of 9.7 months before caregiver report of symptoms (44). This study highlights the need for clinicians to carefully evaluate any deviations in growth curves, particularly in younger patients for whom slowed growth trajectory may be a red flag for disordered eating.

## Catch-up growth is possible but not guaranteed

Deceleration or stunting of linear growth secondary to malnutrition is not irreversible, and adequate nutritional rehabilitation can result in accelerated linear growth (20, 21). A large meta-analysis by Neale et al. (20) analyzed 27 studies regarding growth and puberty in young people with eating disorders. The authors found several unifying and important trends: Growth delay and height stunting are common among patients with restrictive eating disorders, though catch-up growth is possible. Even with catch-up growth, predicted adult height is often not obtained. In most cases weight gain is significantly associated with catch-up growth.

In a study of 46 premenarchal birth-assigned females with eating disorders, maximal growth stunting was noted following 1 year of treatment, even with associated weight gain (36). Catch-up growth was not achieved until 2–4 years from treatment initiation, and weight restoration is strongly predictive of catch-up growth (36). Interestingly, linear growth velocity does not increase during weight restoration, but rather linear growth seems to evolve over a longer duration (36, 45). Armed with this knowledge, clinicians should emphasize the dire importance of aggressive weight restoration and motivate patients and families away from periods of weight stagnation during recovery, as this may lead to irreversible growth stunting.

Small sample-sizes and underrepresentation of birth-assigned males in eating disorder research continues to impede efforts to create sex-specific clinical guidelines. Nonetheless, one study (*n* = 46) of birth-assigned males with eating disorders who displayed growth stunting revealed interesting sex-specific insight into catch-up growth. Those who had not yet started their pubertal growth spurt showed signs of catch-up growth during the first year of treatment with weight gain and did return to premorbid growth curves (46). Conversely, those with previously initiated pubertal growth spurts were not able to return to predicted growth patterns, despite weight gain (46). Even so, clinicians must emphasize that weight restoration is still crucial for bone mineral accretion and the prevention of osteopenia and osteoporosis (47).

Epidemiologic studies suggest the age of onset of AN is decreasing (48, 49). While younger age is a risk factor for poor catch-up growth, delayed bone age may present an opportunity for longer duration of catch-up growth and is an independent predictor of catch-up growth (21, 50). Close monitoring and aggressive weight restoration, even as eating disorder symptoms or behaviors improve, can provide the scaffolding to help patients achieve full linear growth potential. Bone age evaluation can provide a projection for remaining duration of linear growth, though obtaining this evaluation for this purpose is not yet standard of care (51).

## Special considerations in ARFID

Owing to its heterogeneous diagnostic criteria and relatively recent nosology, few studies guide evidence-based medical management and growth implications for patients with Avoidant/Restrictive Food Intake Disorder (ARFID). Introduced in the DSM-5 in 2013, ARFID evolved from prior diagnoses of feeding disorders and results in malnutrition related to sensory aversion, feared consequences of food intake, or low appetite, and in many cases, overlap between these categories (52–54). Patients with ARFID lack the body image distortion that necessarily defines AN. These patients may present to other subspecialist providers for failure to thrive, which can lead to a delay in diagnosis and evidence-based treatment, thus prolonging the course of illness and period of malnutrition. Additionally, those with feeding concerns since infancy or early childhood may have growth curves that do not clearly display growth stunting or deviation. Indeed, patients with ARFID are less likely to present with acute weight loss and associated medical symptoms, like bradycardia (55). Owing to less acute growth deviation and the lack of weight and shape concerns, these patients may avoid medical detection.

Documenting mid-parental height is critical for this population who may not be on track for genetic height potential but otherwise do not have red flags for malnutrition on growth curves alone. Clinicians must set a target weight that is high enough that patients progress through puberty as expected and grow according to their genetic height potential while also accounting for typical child and adolescent growth. Unlike in anorexia nervosa, this target weight may not represent “weight restoration” but rather new weight gain due to chronic malnutrition (56).

Patients with ARFID represent approximately 5–23% of the patients that present to eating disorder programs, and approximately 12% go on to develop AN (57–59). Despite a unifying lack of weight and shape concerns on diagnosis, the etiology and presentation of ARFID remains extremely heterogeneous, as do the neurobiological underpinnings (60). Even as evidence-based treatments emerge, behavioral treatment must be uniquely tailored for each patient. Nonetheless, early and aggressive weight gain and restoration of genetic height potential, as in the other restrictive eating disorders, remains a priority (56).

## Summary of recommendations for providers

• Annual physical exams are critical points of screening for restrictive eating behaviors, even if growth suppression is not yet apparent.
• Providers may choose to conceptualize growth stunting as “covert tissue injury” to appropriately emphasize the ramifications of this medical complication and need for aggressive treatment.
• Any deviation from expected growth trajectory (weight, BMI, and/or length) warrants further medical and psychological evaluation. Children should be growing, and time is of the essence when growth deviations are detected.
• Providers should calculate and document mid-parental height as another marker of growth deviation and as a tool to monitor adequacy of catch-up growth against genetic potential.
• “Normal” weight does not exclude the presence of medical and psychological compromise from an eating disorder.
• Early diagnosis, aggressive nutritional rehabilitation, and frequent reevaluation of target weight to account for expected weight gain during normal growth and development provides the best opportunity for patients to reach their genetic height potential.
• Bone age evaluation can provide a projection for remaining duration of linear growth.
• When catch-up growth is no longer possible, providers must emphasize that weight restoration is still crucial for bone mineral accretion and the prevention of osteopenia and osteoporosis, as well as improved psychological outcomes.
• While there may be sex-specific differences in catch-up growth, aggressive weight restoration portends best chance of catch-up growth for all youth.
• Referral to eating disorder specialists or close collaboration with a multidisciplinary eating disorder team provides the necessary medical, nutritional, and psychological support to restore health.

## Discussion

The impact of restrictive eating disorders on growth and development cannot be overstated, particularly in younger patients. While many consequences of malnutrition are reversible, the loss of genetic height potential in concert with bone demineralization are specific complications which may prove irreversible without early and aggressive weight restoration. Timely intervention is critical and requires providers to identify and explore any deviations in expected growth and development immediately. Once an eating disorder is identified, providers must manage acute complications of malnutrition while also reminding patients and caregivers of those longer-term complications which still require expeditious intervention. Frequent medical visits create the necessary scaffolding to empower caregivers and patients to prioritize early and aggressive weight restoration to bring safely restore medical and psychological wellbeing, in the present and for the future.

## Author contributions

AT conceptualized the review. AD and AR drafted the initial manuscript. AT, AD, and AR critically reviewed and revised the manuscript and approved the final manuscript as submitted. All authors contributed to the article and approved the submitted version.
